# Hydration status in adults with metabolic disorders in relation to socioeconomic, lifestyle and health factors

**DOI:** 10.1371/journal.pone.0305540

**Published:** 2024-07-05

**Authors:** Joanna Frąckiewicz, Anna Ciecierska, Agnieszka Białkowska, Małgorzata Drywień, Jadwiga Hamulka

**Affiliations:** Department of Human Nutrition, Institute of Human Nutrition Sciences, Warsaw University of Life Sciences (WULS-SGGW), Warsaw, Poland; Bay Area Hospital, North Bend Medical Center, UNITED STATES

## Abstract

**Introduction:**

Adequate hydration is essential for maintaining the health and functionality of the human body. This study aimed to examine the association between selected socioeconomic, lifestyle, and health factors and the hydration status of adults with metabolic disorders by analyzing their urine osmolality.

**Methods:**

The study involved 290 adults aged 18–70 years with metabolic disorders. Separate multivariate logistic regression models were conducted to evaluate the factors associated with urine osmolality in tertiles for women and men. Odds Ratios (OR) and 95% Confidence Intervals (95% CI) were calculated.

**Results:**

In women, the following factors of urine osmolality were identified in 1^st^ tertile: age (OR:1.04), physical activity (moderate/high vs. no/low; OR:0.38), and headaches (no vs. yes; OR:1.55), in 2^nd^ tertile: physical activity (moderate/high vs. no/low; OR:2.46) and fatigue during the day (sometimes vs. never/very rarely; OR:0.45), and in 3^rd^ tertile: age (OR:0.94), professional status (‘I work part-time/I study and I work’ vs. ‘I do not work/I study’; OR:0.27), fatigue during the day (very often vs. never/very rarely; OR:2.55), and headaches (no vs. yes; OR:0.44). In men, the following factors of urine osmolality were identified in 1^st^ tertile: place of residence (city vs. village; OR:2.72) and health assessment (average vs. poor; OR:0.32).

**Conclusion:**

Different factors affecting urine osmolality have been identified in women and men. These results highlight the need to implement studies to clarify the relationship between socioeconomic, lifestyle and health factors, and hydration status in adults with metabolic disorders.

## Introduction

Water is the main component of the human body; total body water averages about 60% of the body mass in adult men and 50–55% in women. Therefore, water is a fundamental substance for the human body because it performs many essential functions. First of all, it is a component of all cells and body fluids; it is the environment for all biochemical reactions taking place in the body; it can also be a substrate or product resulting from these reactions. In addition, water plays a vital role in the body’s thermoregulation and excretion of metabolic end products through urine. It also serves a protective and moisturizing function and guarantees the preservation of cognitive performance. Therefore, it is imperative to understand the importance of hydration for human health, as even a slight degree of dehydration is an undesirable condition [1–4].

Dehydration may result from pathological fluid loss and/or reduced water consumption. Limited fluid intake can be caused by many factors, including physical limitations such as reduced mobility or urinary incontinence, social isolation or dementia, and other conditions [5]. The elderly, hospitalized patients and children are more likely to become dehydrated than healthy young adults. This may be due to reduced thirst, viral infections, aging of the body, and the use of diuretics and laxatives [6–8]. Chronic dehydration causes fluid deficits within cells. Any decrease in the stored body water lowers the plasma volume, which can decrease the stroke volume and force the body to compensate by raising the heart rate. A lower plasma volume impacts sweating and blood flow to the skin and, therefore, impedes thermoregulation. When fluid deficiency exceeds 8%, death can occur. Before it reaches this extreme state, dehydration exhibits various symptoms like depressed mood and headache [9–11].

One of the markers used to assess the hydration status is urine osmolality. Urine osmolality is the concentration of osmotic solutes present in the urine and depends on two parameters: the quantity of solutes and the volume of water. Among urinary indicators of hydration status, urinary osmolality is considered one of the more commonly valid techniques. Moreover, urine osmolality reflects dehydration more accurately than blood indices [12–14].

Few studies have shown an association between low usual fluid intake and metabolic disorders, including overweight/obesity, hypertension, diabetes, as well as urolithiasis, constipation, asthma, cardiovascular disease, and some cancers; however, it is not clear if this is a causal relationship [9, 15, 16]. Observational studies have linked habitual low water intake with increased future risk for adverse cardiovascular events, and chronic systemic hypohydration is a proposed pathogenic factor for hypertension. While it is currently unclear how chronic reductions in water intake may predispose individuals to a greater future risk for adverse cardiovascular events, there is evidence that acute hypohydration impairs vascular function and blood pressure regulation [17]. This is disturbing because cardiovascular disease is the leading cause of death in the world. Inadequate hydration assessed by urine biomarkers was associated with elevated BMI and overweight or obesity among adults. This relationship suggests that water, as an essential nutrient, may deserve greater focus in weight management research and clinical strategies. It is widely known that obesity is associated with various comorbidities, including type 2 diabetes, hypertension, coronary artery disease, arterial diseases, and various types of cancer [18–20]. Water is an important ingredient that can be used in treating and preventing diabetes and its complications; therefore, it is crucial for people with diabetes to have adequate fluid intake [21, 22]. Moreover, adequate hydration status is associated with a lower risk of chronic kidney diseases or a slower decline in kidney function and a reduced risk of urinary tract infections, fatal coronary heart disease, and venous thromboembolism [23, 24]. The most effective and cost-effective approach for preventing kidney stones is still high fluid intake, particularly water [25].

Dehydration results in a worse quality of life, especially in hospitalized patients; hence early diagnosis, understanding of the conditions, and implementation of treatment and preventive measures are very crucial. Consequently, the study aimed to identify socioeconomic, lifestyle, and health factors that may contribute to an increased risk of dehydration among adults due to metabolic disorders.

## Materials and methods

### Study design and participants

The study was conducted among 170 women and 120 men, aged 18–70 years old, with diagnosed metabolic disorders. It was designed as a cross-sectional study with convenience sampling. Recruitment lasted from 21/04/2017 to 11/02/2020. The study group was recruited from patients treated in the Metabolic Diseases Outpatient Clinic of the Czerniakowski Hospital in Warsaw. Patients came from the Warsaw agglomeration and other towns from the entire territory of Poland. They were participants diagnosed with the following: diabetes, hypertension, overweight/obesity, circulatory failure, pressure surges, elevated sugar level, and/or dyslipidemia.

**Fig 1:**
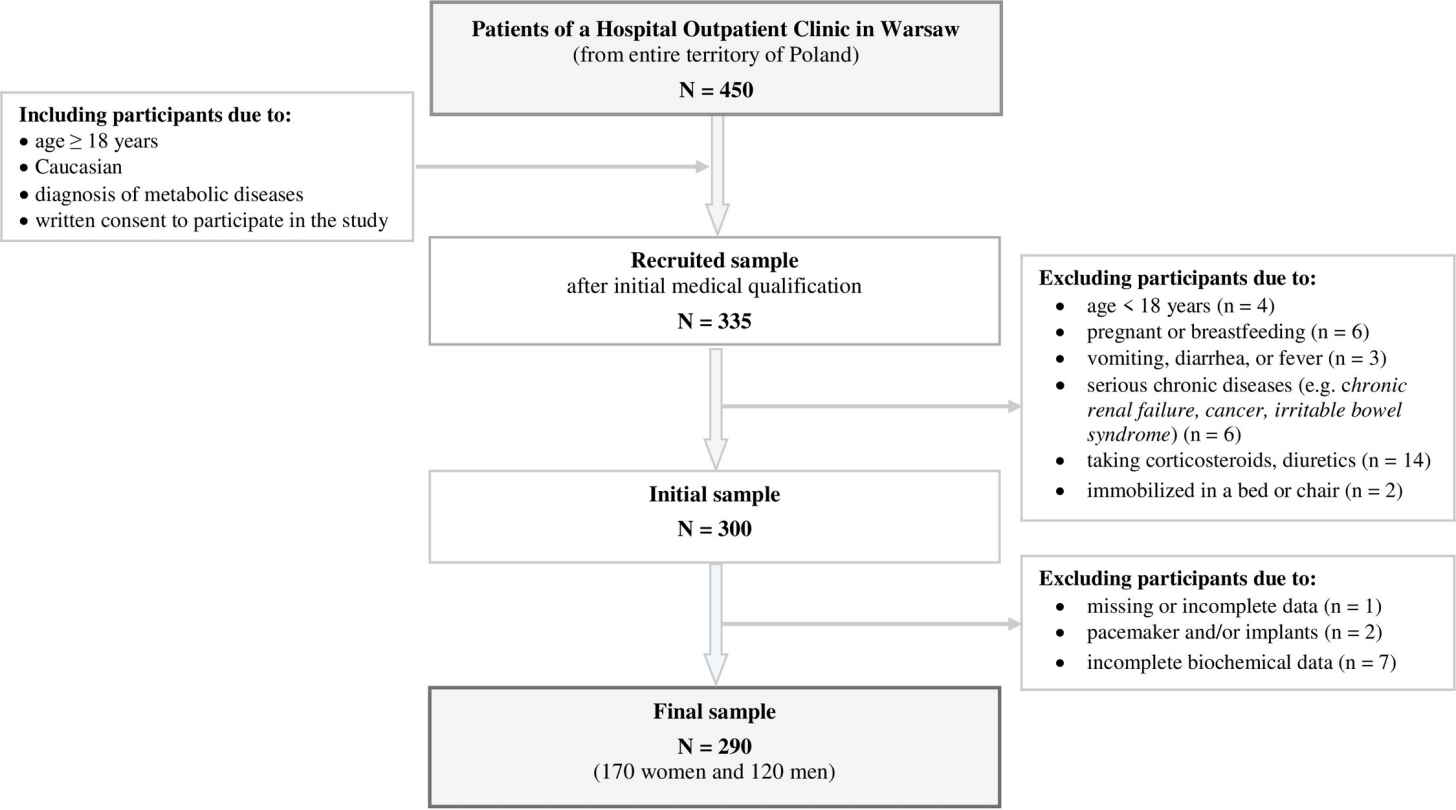
Flowchart: Study design and data collection.

Inclusion criteria were: age 18–70 years, Caucasian, first consultation at the outpatient clinic towards the diagnosis of metabolic syndrome, and conscious and handwritten consent to participate in the study. Exclusion criteria included: lack of consent to participate in the study, diagnosed acute or chronic renal failure, cancer, irritable bowel syndrome, taking corticosteroids, diuretics, and other medications that may affect the results of this study; vomiting, diarrhea, or fever in the last 3 days or immobilized in a bed or chair, pregnant or breastfeeding, and missing or incomplete data of participants. Detailed information on sample recruitment and the methods and procedures used has been published previously [26]. The flowchart depicting the recruitment and retention of participants throughout the study is shown in Fig 1.

### Ethical considerations

The study was conducted according to the Declaration of Helsinki and approved by the Ethics Committee of the Faculty of Human Nutrition and Consumer Science, Warsaw University of Life Sciences, Warszawa, Poland (Resolution No. 04p/2017). The study was conducted according to the guidelines of the Declaration of Helsinki. Before the interview began, the interviewer explained the purpose of the study. All participants gave written informed consent to participate in the study.

### Socioeconomic, lifestyle and health data

General information about the respondents was collected using the survey method. The questionnaire included questions about sex, age, and selected socioeconomic, lifestyle and health factors. Respondents were asked about the occurrence of ailments, i.e., fatigue during the day (yes, no), headaches (yes, no), excessive sweating (yes, no), feeling of fullness and an expanding abdomen (yes, no), heaviness in the legs (yes, no), swelling of the face and eyelids (yes, no), hand swelling (yes, no), swelling of the legs and ankles (yes, no), lower urinary tract infections (yes, no), oliguria (yes, no), very frequent urination (yes, no), and they were asked about feeling thirsty frequently (yes, no), possibility of satisfying the thirst (yes, no), and limitations in the consumption of non-alcoholic beverages (yes, no).

One of the first symptoms of dehydration is thirst. When dehydrated, the human body feels thirsty and will consume fluids until it feels full. In addition, consumed fluids in the mouth and throat provide a sensory stimulus to the body that tells it how thirsty it is. Therefore, the study used a measure of thirst. One of the methods used to determine the subjective measure of thirst is a 7-point scale on which the feeling of thirst at a given moment or most of the time can be marked. The person can choose between the following answers 1 ‐ not at all thirsty, 2 ‐ not thirsty, 3 ‐ not very thirsty, 4 ‐ neutral, 5 ‐ thirsty, 6 ‐ very thirsty, 7 ‐ very, very thirsty. The authors used the thirst scale to make a preliminary assessment of the subjects’ hydration status [27].

### Non-alcoholic beverages consumption data

The study used the Dietary Habits and Nutrition Beliefs Questionnaire (KomPAN) to assess the frequency of consumption of selected non-alcoholic beverages [28–30]. Beverages consumption data was collected using a semi-quantitative food frequency questionnaire (FFQ) in the last three months according to the following categories: never; <1 serving/month; 1–3 servings/month; 1–2 serving/week; 3–4 servings/week, 5 servings/week; 1 serving/day; 2 servings/day; ≥ 3 servings/day. Information was collected from the respondents on the frequency of consumption of non-alcoholic beverages, i.e., tea, coffee, cereal coffee, milk, fermented milk drinks, cocoa, drinking chocolate, mineral water, pure tap water, juices, non-carbonated fruit drinks, fruit nectars, sweetened sparkling drinks, tea drinks, cola drinks, energy drinks, isotonic drinks, compotes, diluted syrups, non-alcoholic beers. Subjects completed the FFQ in the presence of the same person ‐ a well-trained dietician and hospital staff member. This was the basis for eliminating errors and standardizing the procedure for completing the questionnaire by all respondents. In addition, direct contact with the dietitian helped to clarify any doubts about how to complete the questionnaire.

Information on the size of the usually consumed portion was also collected: for a drink ‐ a 150 ml cup, a glass ‐ 250 ml, a 300 ml mug; for dairy products: kefir, buttermilk, yoghurt ‐ package size; for other beverages ‐ a can, a bottle. Then, the daily beverage intake (ml/d) was calculated and compared with reference values for an adequate intake (AI = 2000 ml for women and 2500 ml for men) [31]. In order to estimate the daily consumption of each beverages, data from the FFQ were used, i.e. the frequency of consumption and the consumed portion size (in ml) of each drink [28–30].

### Hydration status

The hydration status was tested by measuring urine osmolality (mOsm/kg). Urine samples were collected on an ongoing basis from 8/05/2017 to 15/02/2020. The collected samples were coded and their identification was not possible. Urine was collected in the morning, including discarding the first fraction. Urine samples were secured (marked) and stored frozen (−20°C) for further analysis [32]. Urine osmolality was determined by a freezing-point osmometer (cryoscopic method) (Marcel OS3000 osmometer, Warsaw, Poland).

### Statistical analysis

The results are reported as means ±standard deviation (SD) and range or percentages. Differences between groups were tested with the Chi-square test (categorical variables) or the U-Mann-Whitney test (continuous variables). Before statistical analysis, the normality of variable distribution was checked with a Kolmogorov–Smirnov test.

Firstly, according to the urine osmolality, the participants were divided into two groups based on their hydration statuses as follows: a dehydrated status and normal hydration status. A dehydrated status was defined as urine osmolality >800 mOsm/kg [33, 34]. In our study, it was decided to divide urine osmolality into tertiles because different cut-offs for dehydration have been used in the literature [20, 35–37]. In addition, the study found a relatively small percentage of people who were dehydrated after the cut-off >800 mOsm/kg H_2_O, and the division into tertiles allowed obtaining three equal groups of subjects. Therefore, to explore the associations between urine osmolality and selected factors, two logistic regression models (odds ratios, ORs with 95% confidence intervals, 95% CIs) were calculated: age-adjusted (Model 1) and multivariate-adjusted (Model 2). Multivariate logistic regression models were conducted to evaluate factors related to urine osmolality in tertiles. Results were presented by tertiles of urine osmolality separately for women and men. For women, the cut-off values were <390 mOsm/kg, 390–615 mOsm/kg, and >615 mOsm/kg; for men were <449 mOsm/kg, 449–633 mOsm/kg, and >633.0 mOsm/kg.

Multivariate-adjusted models included selected factors, i.e., the age of participants (continuous variable), level of education (primary/professional, secondary/’I study’, or higher), place of residence (village, town, or city), professional status (‘I do not work/I study’, ‘I work part-time/I study and I work’, or ‘I work full-time’), self-reported economic status (very poor/poor, average, or very good), self-reported physical activity level (no/low, moderate/high), self-reported health status (poor, average, or good/very good), fatigue during the day (never/very rarely, sometimes, or very often), headaches (yes, no), smoking cigarettes (yes, no), consumption of non-alcohol beverages (yes, no).

Results were considered significant when p < 0.05. Data were analyzed with STATISTICA 13.3 computer software (TIBCO Software Inc., StatSoft, USA).

## Results

Table 1 shows the characteristics of the study population. Of the 290 individuals, 120 (41.4%) were men, and 170 (58.6%) were women, adults with diagnosed metabolic disorders. The mean age was 53.1±12.9 years. We observed that a statistically significant higher number of men had primary or secondary education than women. We also found that significantly more women used dietary supplements compared to men. The study found no significant association between the mean consumption of non-alcoholic beverages or mean urine osmolality between women and men.

**Table 1:** Baseline characteristics of the study population.

Variables	Total	Women	Men	p-value
	n = 290	n = 170	n = 120	
**Age (years)**	53.1±12.9^a^	52.5±13.5	54.0±12.1	0.477^c^
	56.0^b^	56.0	56.0	
**Education (%)**				
Primary or secondary	23.2	17.1	29.2	0.007^d^
Higher	76.9	82.9	70.8	
**Place of residence (%)**				
Village	9.3	7.7	10.8	0.644^d^
Town	15.4	15.8	15.0	
City	75.3	76.5	74.2	
**Physical activity (%)**				
Non or low	74.6	74.1	75.0	0.312^d^
Moderate or high	25.5	25.9	25.0	
**Economic status (%)**				
Very poor or poor	10.1	11.0	9.2	0.375^d^
Good	52.6	57.7	47.5	
Very good	37.3	31.3	43.3	
**Medicine use (%)**	88.9	89.4	88.3	0.772^d^
**Supplement use (%)**	18.3	28.2	8.3	<0.001^d^
**Special diet (%)**	18.8	21.8	15.8	0.208^d^
**Cigarette smoking (%)**	23.4	23.5	23.3	0.969^d^
**Non-alcoholic beverages**	2849±1165	2734±1082	3011±1261	0.070^d^
**(ml/d)**	2523	2427	2700	
**Urine osmolality**	544.0±234.0	524.9±231.6	570.4±237.8	0.139^c^
**(mOsm/kg H**_**2**_ **O)**	524.0	503.0	563.0	

Note:

^a^Mean ± SD (standard deviation)

^b^Median

^c^U-Mann-Whitney test

^d^Chi^2^ Pearson test.

Daily consumption of non-alcoholic beverages was compared with reference values, and it was found that significantly more women (77.1%) compared to men (65.0%) consumed adequate water during the day (p = 0.024).

Table 2 shows diseases associated with metabolic disorders and selected ailments that may be related to hydration status among participants. We observed statistically significantly more men diagnosed with diabetes, overweight, or very frequent urination compared to women. A significant statistical difference was found between men and women, with a higher percentage of women reporting symptoms such as fatigue, headaches, fullness, abdominal expansion, leg heaviness, and swelling in the face, hands, legs, and ankles.

**Table 2:** Diseases and selected ailments among participants (%).

Variables	Total	Women	Men	p-value^a^
	n = 290	n = 170	n = 120	
**Diseases**				
Diabetes	45.9	39.4	52.5	0.027
Hypertension	65.5	65.9	65.0	0.876
Overweight	24.4	17.1	31.7	0.003
Obesity	47.4	46.5	48.3	0.754
Circulatory failure	12.6	11.8	13.3	0.689
Pressure surges	42.0	36.5	47.5	0.060
Elevated sugar	59.5	54.7	64.2	0.107
Dyslipidemia	4.9	4.7	5.0	0.908
**Selected ailments**				
Fatigue during the day	28.7	38.2	19.2	0.007
Headaches	53.7	60.6	46.7	0.039
Excessive sweating	51.0	51.2	50.8	0.954
Feeling of fullness and an expanding abdomen	39.8	47.1	32.5	0.013
Heaviness in the legs	42.9	52.4	33.3	0.001
Swelling of the face and eyelids	14.5	24.7	4.2	<0.001
Hand swelling	16.8	25.3	8.3	<0.001
Swelling of the legs and ankles	34.8	41.2	28.3	0.045
Lower urinary tract infections	5.4	6.5	4.2	0.397
Oliguria	5.9	5.9	5.8	0.986
Very frequent urination	28.3	22.4	34.2	0.026

Note:

^a^Chi^2^ Pearson test.

The characteristics of the total study sample concerning factors connected with feeling thirsty and limitation of beverage consumption are presented in Table 3. Statistically, significantly more women were thirsty or very thirsty at the moment of data collection compared to men.

**Table 3:** Feeling thirsty and limitation of beverages consumption among participants (%).

Variables	Total	Women	Men	p-value^a^
	n = 290	n = 170	n = 120	
**Feeling thirsty usually (SCALE)**				
not at all thirsty/not thirsty	26.2	28.2	24.1	0.659
neutral	41.9	42.9	40.8	
thirsty or very thirsty	31.9	28.9	35.1	
**Feeling thirsty for the moment of the data collection (SCALE)**				
not at all thirsty/not thirsty	46.3	46.0	46.6	0.046
neutral	37.0	34.7	39.2	
thirsty or very thirsty	16.7	19.3	14.2	
**Feeling thirsty frequently**	34.1	32.4	35.8	0.453
**Possibility of satisfying the thirst**	85.8	84.0	87.5	0.708
**Limitations in the consumption of beverages**	4.2	5.9	2.5	0.167

Note:

^a^Chi^2^ Pearson test.

Based on the measurement of urine osmolality, it was found that 14% of the subjects were dehydrated. Table 4 shows the differences between the hydration status of the respondents divided into two groups according to their hydration status as follows: a dehydrated status (>800 mOsm/kg) and normal hydration status (≤800 mOsm/kg) and selected socioeconomic, lifestyle and health factors. It was found that dehydration was characterized by younger people with an average age of 47.2 compared to people with a normal state of hydration, where the average age was over 54 years. It was also shown that the percentage of dehydrated people who declared full-time employment was significantly the highest compared to other professional statuses (Table 4).

**Table 4:** Differences between hydration status and selected socioeconomic, lifestyle and health factors.

Variables	Normal hydration status^a^	Dehydrated status^b^	p-value
	n = 249	n = 41	
**Age (years)**	54.1 ± 12.6^c^	47.2 ± 13.4	0.002^e^
	57.0^d^	48.0	
**Sex**			
Women	59.0^e^	56.1	0.723^f^
Men	41.0	43.9	
**Education**			
Primary/professional	22.9	17.1	
Secondary/’I study’	41.8	53.7	0.357^f^
Higher	35.3	29.3	
**Place of residence**			
Village	8.8	9.8	
Town	14.5	21.5	0.441^f^
City	76.7	68.3	
**Professional status**			
‘I do not work/I study’	44.6	24.4	
‘I work part-time/I study and I work’	9.2	9.8	0.044^f^
‘I work-full time’	46.2	65.9	
**Economic status**			
Very poor/poor	10.8	7.3	
Average	53.4	53.7	0.768^f^
Very good	35.7	39.0	
**Physical activity**			
No/low	73.9	78.1	0.572^f^
Moderate/high	26.1	21.9	
**Health status**			
Poor	17.7	12.2	
Average	58.2	73.2	0.191^f^
Good/very good	24.1	14.6	
**Fatigue during the day**			
Never/very rarely	19.3	17.1	
Sometimes	49.8	48.8	0.899^f^
Very often	30.9	34.2	
**Headaches**			
Yes	53.4	63.4	0.233^f^
No	46.6	36.6	
**Cigarette smoking**			
Yes	23.3	24.4	0.878^f^
No	76.7	75.6	
**Non-alcoholic beverages**			
≤ Median^g^	49.8	51.2	0.866^f^
> Median	50.2	48.8	

Note:

^a^Urine osmolality ≤ 800 mOsm/kg H_2_O

^b^Urine osmolality > 800 mOsm/kg H_2_O

^c^Mean ± SD (standard deviation)

^d^Median

^e^U-Mann-Whitney test

^f^Chi^2^ Pearson test

^g^Median: 2523 ml.

Multivariate-adjusted odds ratios (ORs) of urine osmolality in tertiles by socioeconomic, lifestyle, and health factors in adults with metabolic disorders are presented in Tables 5 and 6, separately for women and men. With increasing age, women were more likely to be in the 1^st^ tertile of urine osmolality and less likely to be in the 3^rd^ tertile of urine osmolality (OR:1.04, 95% CI:1.00–1.07 and OR:0.94, 95% CI:0.91–0.97, respectively). The likelihood of women being in the 1^st^ (lowest) osmolality tertile was reduced for those who had moderate/high physical activity (OR:0.38, 95% CI:0.16–0.91) compared to those who had no/low activity (Table 5). In the same osmolality tercile, women were more likely to report no headaches (OR:1.55, 95% CI:1.11–3.07) compared to those with headaches. Individuals with moderate to high physical activity were more likely to be in the 2^nd^ osmolality tertile (OR:2.46, 95% CI:1.15–3.29) compared to those with low or no activity. Conversely, those who occasionally experience fatigue during the day were less likely to be in the 2^nd^ osmolality tertile (OR:0.45, 95% CI:0.17–0.96) compared to those who never or very rarely experience fatigue during the day. Women who work part-time/work and study were found to be less likely to be in the 3^rd^ (highest) osmolality tertile (OR: 0.27, 95% CI: 0.06–0.99) compared to those who do not work/’I study’. Similarly, those who did not experience headaches were less likely to be in the highest tertile (OR: 0.44, 95% CI: 0.21–0.96) compared to those who reported having headaches. Conversely, women who experienced frequent fatigue during the day were more likely to be in the 3^rd^ osmolality tertile (OR: 2.55, 95% CI: 1.69–3.39) than those who never or rarely experienced fatigue during the day. For men, those who lived in cities were more likely to be in the 1^st^ (lowest) osmolality tertile (OR: 2.72, 95% CI: 1.05–3.52) compared to those who lived in villages. On the other hand, those who declared an average health status were less likely to be in the lowest osmolality tertile (OR: 0.32, 95% CI: 0.10–0.97) compared to those who declared bad health status (Table 6). Moreover, individuals who perceived their health status as average were more likely to be in the 3^rd^ (highest) osmolality tertile (OR: 2.25, 95% CI: 1.12–3.29) compared to those who declared poor health status.

**Table 5:** The logistic regression of urine osmolality in tertiles and socioeconomic, lifestyle and health factors in women (n = 170).

Variables	1^st^ Tertile^a^		2^nd^ Tertile^b^		3^rd^ Tertile^c^	
	Model 1^d^	Model 2^e^	Model 1	Model 2	Model 1	Model 2
	OR (95% CI)^f^	OR (95% CI)	OR (95% CI)	OR (95% CI)	OR (95% CI)	OR (95% CI)
**Age (years)**	1.03 (1.01–1.06)	1.04 (1.00–1.07)	1.03 (1.01–1.06)	1.02 (0.98–1.06)	0.94 (0.92–0.97)	0.94 (0.91–0.97)
p for trend	0.019	0.033	0.020	0.196	< 0.001	< 0.001
**Education**						
Primary/professional	1.00	1.00	1.00	1.00	1.00	1.00
Secondary/’I study’	0.67 (0.27–1.66)	0.67 (0.26–1.75)	0.96 (0.40–2.31)	1.02 (0.41–2.52)	1.54 (0.61–3.92)	1.51 (0.53–3.36)
Higher	1.27 (0.50–3.19)	1.47 (0.54–3.97)	0.70 (0.28–1.78)	0.83 (0.31–2.17)	1.13 (0.42–3.01)	0.81 (0.26–2.53)
**Place of residence**						
Village	1.00	1.00	1.00	1.00	1.00	1.00
Town	1.40 (0.30–3.49)	1.38 (0.28–3.83)	1.55 (0.38–3.31)	1.37 (0.32–2.82)	0.49 (0.13–1.93)	0.47 (0.10–2.16)
City	1.71 (0.45–3.52)	1.40 (0.35–2.65)	1.15 (0.34–3.95)	0.89 (0.25–3.19)	0.56 (0.18–1.76)	0.79 (0.23–2.76)
**Professional status**						
‘I do not work/I study’	1.00	1.00	1.00	1.00	1.00	1.00
‘I work part-time/I study and I work’	0.56 (0.17–1.91)	1.03 (0.27–3.88)	1.69 (0.57–2.99)	2.29 (0.80–3.07)	0.96 (0.28–3.34)	0.27 (0.06–0.99)*
‘I work full-time’	0.69 (0.35–1.35)	1.20 (0.53–2.74)	0.89 (0.55–1.75)	1.13 (0.51–2.53)	2.11 (1.07–2.77)*	0.81 (0.35–1.89)
**Economic status**						
Very poor/poor	1.00	1.00	1.00	1.00	1.00	1.00
Average	1.36 (0.45–2.09)	1.40 (0.44–2.39)	0.67 (0.24–1.82)	0.67 (0.24–1.85)	1.15 (0.40–2.30)	1.19 (0.37–2.88)
Very good	1.44 (0.45–2.63)	1.63 (0.48–2.56)	0.77 (0.26–2.24)	0.90 (0.30–2.70)	0.94 (0.30–2.90)	0.73 (0.20–2.64)
**Physical activity**						
No/low	1.00	1.00	1.00	1.00	1.00	1.00
Moderate/high	0.37 (0.16–0.87)*	0.38 (0.16–0.91)*	1.86 (1.01–3.76)*	2.46 (1.15–3.29)*	1.23 (0.60–2.52)	0.95 (0.42–2.15)
**Health status**						
Poor	1.00	1.00	1.00	1.00	1.00	1.00
Average	0.63 (0.27–1.46)	0.69 (0.29–1.65)	0.83 (0.36–1.89)	0.95 (0.41–2.23)	2.03 (0.79–3.13)	1.69 (0.61–2.66)
Good/very good	0.98 (0.36–2.66)	0.95 (0.34–2.81)	0.76 (0.27–2.10)	0.89 (0.31–2.51)	1.43 (0.47–2.38)	1.17 (0.34–2.03)
**Fatigue during the day**						
Never/very rarely	1.00	1.00	1.00	1.00	1.00	1.00
Sometimes	1.17 (0.45–2.04)	1.47 (0.53–2.07)	0.45 (0.18–0.97)*	0.45 (0.17–0.96)*	2.20 (1.02–3.49)*	2.25 (0.62–3.18)
Very often	0.86 (0.32–2.32)	1.01 (0.35–2.87)	0.62 (0.24–1.57)	0.61 (0.23–1.58)	2.14 (1.71–3.45)*	2.55 (1.69–3.39)*
**Headaches**						
Yes	1.00	1.00	1.00	1.00	1.00	1.00
No	1.62 (1.02–3.11)*	1.55 (1.11–3.07)*	1.35 (0.71–2.56)	1.21 (0.62–2.34)	0.44 (0.22–0.88)*	0.44 (0.21–0.96)*
**Cigarette smoking**						
Yes	1.00	1.00	1.00	1.00	1.00	1.00
No	1.35 (0.62–2.95)	1.43 (0.63–3.21)	0.98 (0.47–2.07)	0.98 (0.46–2.10)	0.77 (0.37–1.61)	0.69 (0.31–1.56)
**Non-alcoholic beverages**						
≤ Median	1.00	1.00	1.00	1.00	1.00	1.00
> Median	0.76 (0.40–1.46)	0.71 (0.27–1.88)	0.95 (0.51–1.79)	1.30 (0.51–2.35)	1.38 (0.73–2.62)	1.10 (0.39–3.07)

Note:

^a^1^st^ Tertile: < 390 mOsm/kg H_2_O

^b^2^nd^ Tertile: 390–615 mOsm/kg H_2_O

^c^3^rd^ Tertile: > 615 mOsm/kg H_2_O

^d^Model 1: age-adjusted model

^e^Model 2: multivariate-adjusted model

^f^OR: odds ratio; CI: confidence interval; Median: 2427 ml

* p-value ≤ 0.05.

**Table 6:** The logistic regression of urine osmolality in tertiles and socioeconomic, lifestyle and health factors in men (n = 120).

Variables	1^st^ Tertile^a^		2^nd^ Tertile^b^		3^rd^ Tertile^c^	
	Model 1^d^	Model 2^e^	Model 1	Model 2	Model 1	Model 2
	OR (95% CI)^f^	OR (95% CI)	OR (95% CI)	OR (95% CI)	OR (95% CI)	OR (95% CI)
**Age (years)**	1.05 (1.01–1.08)	1.05 (0.99–1.10)	1.01 (0.98–1.04)	1.00 (0.95–1.04)	0.96 (0.93–0.99)	0.97 (0.93–1.01)
p for trend	0.019	0.081	0.689	0.885	0.007	0.129
**Education**						
Primary/professional	1.00	1.00	1.00	1.00	1.00	1.00
Secondary/’I study’	1.09 (0.42–2.81)	1.29 (0.49–3.43)	0.75 (0.30–1.88)	0.75 (0.29–1.93)	1.25 (0.48–3.27)	1.04 (0.38–2.88)
Higher	1.05 (0.40–2.78)	1.26 (0.45–3.57)	0.64 (0.25–1.67)	0.58 (0.21–1.60)	1.50 (0.57–3.97)	1.41 (0.49–2.07)
**Place of residence**						
Village	1.00	1.00	1.00	1.00	1.00	1.00
Town	1.10 (0.16–3.74)	1.50 (0.20–3.17)	0.33 (0.07–1.48)	0.38 (0.08–1.78)	2.81 (0.63–3.61)	1.92 (0.40–3.34)
City	2.40 (1.01–3.28)*	2.72 (1.05–3.52)*	0.41 (0.13–1.34)	0.41 (0.12–1.38)	0.93 (0.26–3.28)	0.87 (0.23–2.28)
**Professional status**						
‘I do not work/I study’	1.00	1.00	1.00	1.00	1.00	1.00
‘I work part-time/I study and I work’	0.57 (0.13–2.47)	0.80 (0.17–2.70)	0.87 (0.22–2.45)	0.77 (0.18–2.21)	2.16 (0.52–3.03)	1.76 (0.38–2.13)
‘I work full-time’	0.62 (0.28–1.39)	1.08 (0.41–2.82)	0.67 (0.30–1.49)	0.66 (0.25–1.72)	2.62 (1.08–3.33)*	1.59 (0.57–2.41)
**Economic status**						
Very poor/poor	1.00	1.00	1.00	1.00	1.00	1.00
Average	0.68 (0.18–2.65)	0.61 (0.15–2.52)	0.81 (0.21–3.11)	0.79 (0.20–3.09)	1.81 (0.43–3.53)	2.30 (0.50–3.73)
Very good	1.01 (0.26–2.89)	1.05 (0.25–2.31)	1.01 (0.26–3.89)	0.96 (0.24–3.74)	0.98 (0.23–2.24)	1.05 (0.22–2.01)
**Physical activity**						
No/low	1.00	1.00	1.00	1.00	1.00	1.00
Moderate/high	0.55 (0.21–1.43)	0.70 (0.26–1.91)	0.95 (0.40–2.28)	1.05 (0.42–2.62)	1.78 (0.76–3.18)	1.32 (0.53–2.29)
**Health status**						
Poor	1.00	1.00	1.00	1.00	1.00	1.00
Average	0.37 (0.13–0.98)*	0.32 (0.10–0.97)*	1.11 (0.37–2.32)	1.23 (0.40–2.82)	2.96 (1.08–3.18)*	2.25 (1.12–3.29)*
Good/very good	0.52 (0.16–1.70)	0.53 (0.15–1.85)	0.91 (0.27–2.12)	0.95 (0.27–2.44)	2.62 (0.62–3.04)	2.75 (0.59–3.87)
**Fatigue during the day**						
Never/very rarely	1.00	1.00	1.00	1.00	1.00	1.00
Sometimes	0.96 (0.37–2.48)	1.22 (0.45–3.33)	0.67 (0.27–1.64)	0.68 (0.27–1.73)	1.56 (0.62–3.92)	1.24 (0.45–3.37)
Very often	1.83 (0.60–3.57)	2.03 (0.64–3.48)	0.84 (0.28–2.52)	0.96 (0.31–2.98)	0.58 (0.17–2.04)	0.41 (0.11–1.62)
**Headaches**						
Yes	1.00	1.00	1.00	1.00	1.00	1.00
No	1.64 (0.75–3.57)	1.40 (0.62–3.16)	(0.48–2.17)	1.02 (0.45–2.21)	0.61 (0.28–1.30)	0.73 (0.32–1.66)
**Cigarette smoking**						
Yes	1.00	1.00	1.00	1.00	1.00	1.00
No	1.27 (0.50–3.21)	1.12 (0.42–2.95)	0.75 (0.31–1.79)	0.71 (0.29–1.75)	1.07 (0.44–2.65)	1.34 (0.50–3.56)
**Non-alcoholic beverages**						
≤ Median	1.00	1.00	1.00	1.00	1.00	1.00
> Median	0.93 (0.43–1.99)	0.76 (0.24–2.44)	1.08 (0.51–2.29)	1.97 (0.62–3.20)	1.00 (0.47–2.14)	0.66 (0.20–2.18)

Note:

^a^1^st^ Tertile: < 449 mOsm/kg H_2_O

^b^2^nd^ Tertile: 449–633 mOsm/kg H_2_O

^c^3^rd^ Tertile: > 633 mOsm/kg H_2_O

^d^Model 1: age-adjusted model

^e^Model 2: multivariate-adjusted model

^f^OR: odds ratio; CI: confidence interval; Median: 2700 ml

* p-value ≤ 0.05.

## Discussion

This study provides interesting insights regarding socioeconomic, lifestyle, and health factors related to osmolality urine among adults diagnosed with metabolic disorders aged 18–70. We did not observe a statistically significant difference in the mean urine osmolality between the examined women and men. Different factors affecting urine osmolality in tertiles have been identified in women and men. The study demonstrates that in women, the following urine osmolality factors were identified in the 1^st^ tertile: age, moderate/high physical activity, and no headaches, in the 2^nd^ tertile: moderate/high physical activity (vs. no/low) and sometimes fatigue during the day (vs. never/very rarely), and in the 3^rd^ teritle: age, professional status as ‘I work part-time/I study and I work’ (vs. ‘I do not work/I study’), frequent fatigue during the day (vs. never/very rarely), and no headaches (vs. yes). In men, the following urine osmolality factors were identified in the 1^st^ tertile: city as place of residence (vs. village) and average self-assessment health (vs. bad).

Our findings indicate that mean urine osmolality in adults with metabolic disorders was 544 mOsm/kg H_2_O. We did not observe a statistically significant difference in the mean urine osmolality between the examined women and men; however, this value was higher among men. It has also been confirmed in other studies in different age groups conducted on healthy individuals and patients, where the typical values ranged from 50 to 1400 mOsm/kg [20, 33–37]. A healthy, dehydrated individual will have a small amount of highly concentrated urine, reflected by elevated urine osmolality. On the other hand, someone with a high fluid intake will produce a large amount of urine, resulting in low urine osmolality. Thus, urine osmolality reflects the capacity of the kidney to appropriately respond to variations in body water balance [1, 38]. Several cut-offs of urine osmolality have been proposed to classify subjects according to their hydration status, such as 500 mOsm/kg [33], 800 mOsm/kg [18] or 850 mOsm/kg [39] but generally hypohydration is defined as urine osmolality greater than 800 mOsm/kg H_2_O [1]. These differences are influenced by unique regional customs involving beverages and food items and the fact that daily human requirement for water increases as sodium and protein intakes increase [12]. Although studies have reported on the assessment of hydration status using urine osmolality in healthy populations, similar assessments are lacking for adults with metabolic disorders. Yeh et al. found that medical conditions, including diabetes and hypertension were not associated with the level of urine osmolality; only participants with chronic kidney disease tended to have lower urine osmolality [35].

In this study, we found that as women age, they are more likely to be in the 1^st^ tertile of urine osmolality and less likely to be in the 3^rd^ tertile of urine osmolality. Similarly, Yeh et al. found that elderly individuals and women tend to have more diluted urine [35]. Their research showed that for every 10-year increase in age, the likelihood of urine over-dilution increased by an adjusted odds ratio of 1.10. Likewise, Perinpam et al. found that in multivariable models, urine osmolality declined with age and remained statistically higher in men than women [36]. However, all older or hospitalized persons should be considered at risk of low-intake dehydration and encouraged to consume adequate amounts of drinks. Generally, interventions shall be individualized, comprehensive, and part of a multimodal and multidisciplinary team approach. A range of effective interventions is available to support adequate nutrition and hydration in older and hospitalized persons to maintain or improve nutritional status, clinical course and quality of life. These interventions should be implemented in clinical practice and routinely used [38].

We discovered that women who worked part-time or worked and studied simultaneously were less likely to be in the 3^rd^ tertile of urine osmolality. This may be due to the fact that women who did not work full-time had a greater opportunity to replenish fluids during the day compared to those in full-time employment who did not have the opportunity to drink adequate amounts of fluids. Similarly, El-Sharkawy et al. found that the average urine osmolality was significantly higher at the end of the shift compared to the beginning in people working full-time [40]. Dehydration in nurses and doctors working in medical and surgical emergency rooms resulting from lower fluid intake during work, was consequently associated with some cognitive impairment in these people [40]. However, in the study by Orysiak et al., no statistically significant differences in urine osmolality were found between the beginning and the end of work in men who worked as foresters [41].

In our study, we observed that women with moderate or high physical activity were less likely to be in the 1st tertile of urine osmolality and more likely to be in the 2^nd^ tertile of urine osmolality. The reason for this may be related to the body losing water through sweat during increased physical activity and not replenishing it adequately. As a result, the urine becomes concentrated, and its osmolality increases. According to Deger et al., among 45 hospital visitors who were interested in participating in the study, it was found that increasing physical activity led to a significant decrease in osmolality at night and over the 24-hour period [42]. However, there was no significant effect of movement on osmolality during the day. The average osmolality for the less active group was 550.4 ± 248.5 mOsm/kg, while the moderate and more active groups had averages of 524.0 ± 256.4 mOsm/kg and 477.7 ± 237.9 mOsm/kg, respectively. Nevertheless, there was no significant difference in osmolality between the three groups [42].

Furthermore, this study found that women who frequently experienced fatigue during the day were more likely to be in the 3^rd^ tertile of urine osmolality, while those who did not report headaches were less likely to be in this tertile. Fatigue and headache are typical symptoms of people who do not drink enough fluids during the day. It is known that insufficient water intake affects the inadequate hydration status of the human body, which in turn affects cognitive functions and human health and is manifested by fatigue during the day or headaches [43].

The obtained results showed that in men, only two factors affecting urine osmolality were identified, i.e., place of residence and self-assessment of health status. Men residing in urban areas were more likely to be in the 1^st^ tertile of urine osmolality. It is possible that men had better access to drinks or water dispensers due to their place of residence, which could have contributed to their higher levels of hydration. This situation could also be related to the greater awareness among urban men of the role of adequate beverage intake during the day. In contrast, men of average health were more likely to be in the 3^rd^ tertile of urine osmolality than in the 1^st^ tertile. This could be attributed to the insufficient intake of fluids by some men, which may have resulted in them feeling marginally worse than reported.

### Strengths and limitations of the study

The strength of our results draw attention to urine as a readily available and appropriable biological material for hydration status. The advantages of measuring urine osmolality, which is considered the gold standard for assessing urine concentration, are manifold. First, it is a cheap and non-invasive method that can be used for individuals and large population groups of different ages. The method enables quick and easy detection of dehydration tendencies as osmolality rises in conjunction with hypertonic dehydration. In addition, the method is sensitive enough to detect slight changes in hydration status. Although the sample does not cover the entire territory of Poland, it reflects Poles’ demographic and social diversity. Additionally, it is crucial to highlight the innovative approach taken in this study, as there are only a few studies in this field related to people with metabolic disorders.

The findings of this study should also be considered within the context of its limitations. This study was conducted with a sample of 290 adults, and some may argue that it not sizeable enough to detect statistical significance. Also, the recruitment methods used in the study did not allow us to calculate the response rate and the reasons for refusals. Thus, the possibility that this sample can be biased towards a more responsive group of adults cannot be ruled out. The research sample is not representative because the study involved people with metabolic disorders who came to the clinic on their own, therefore our research results cannot be generalized to the entire population‥ Moreover, the project’s timeline did not include the participants’ food intake assessment, and thus, no information on food ingestion is available, which could be an additional source of water for the test subjects. Non-alcoholic beverage consumption was collected using the FFQ method, which may result in errors regarding the actual consumption of drinks, but FFQ is a validated and widely used questionnaire.

## Conclusions

Our study revealed different selected socioeconomic, lifestyle and health factors affecting urine osmolality in women and men with diagnosed metabolic disorders. Interestingly, more factors were detected in the group comprising women than men. These factors can cause low beverage intake, leading to high osmolality and consequently dehydration, negatively impacting well-being and quality of life. More insightful and in-depth studies are required to identify the factors that affect hydration status. In addition, understanding the socioeconomic and health situation of hospitalized people is essential for formulating preventive plans adequate to their realities.

## Supporting information

S1 Data (XLSX)

S1 Checklist STROBE statement—checklist of items that should be included in reports of observational studies. (DOCX)
